# A Case of Ventricular Arrhythmia in a Patient With HER2-Positive Gastric Cancer Receiving Trastuzumab

**DOI:** 10.1155/crgm/2752788

**Published:** 2025-07-15

**Authors:** Naoto Takahashi, Hitoshi Fujii, Tomohiro Iwasa, Yuka Koizumi, Yukihiro Minagawa, Chihiro Tono

**Affiliations:** Department of Surgery, Iwate Prefectural Kuji Hospital, Kuji, Iwate, Japan

## Abstract

**Background:** Human epidermal growth factor receptor 2 (HER2)-positive gastric cancer accounts for approximately 15% of gastric cancer cases. Trastuzumab (Trz), a monoclonal antibody targeting HER2, has been shown to improve overall survival when combined with chemotherapy. However, while Trz-induced cardiotoxicity (TIC) is a well-recognized adverse effect in breast cancer chemotherapy, reports on its occurrence in gastric cancer treatment remain limited.

**Case Presentation:** An 80-year-old Japanese male with HER2-positive advanced gastric cancer (cStage III) developed ventricular arrhythmia and heart failure during postoperative chemotherapy with the Trz + SOX regimen (Trz, oxaliplatin, and TS-1). The patient initially underwent distal gastrectomy with D1+ lymphadenectomy for anemia and pyloric stenosis. Metastasis to the #8a lymph node (anterior superior lymph node of the common hepatic artery) and pancreatic invasion via lymph nodes were treated with two cycles of the Trz + SOX regimen, leading to a partial response. However, after the 11th cycle, he developed ventricular tachycardia and heart failure. Cardiac imaging and laboratory findings revealed no coronary artery disease or structural abnormalities, suggesting TIC as the underlying cause. Antiarrhythmic therapy with pharmacological agents led to symptom resolution, and no recurrence of arrhythmia or heart failure was observed.

**Discussion:** This case highlights the potential cardiotoxicity associated with nonanthracycline-based Trz regimens for gastric cancer. Pathophysiologically, HER2 signaling inhibition in cardiomyocytes may impair stress responses and repair mechanisms. The patient's advanced age, history of hypertension and anemia, and cumulative exposure to chemotherapy may have contributed to increased cardiac vulnerability. Careful monitoring of cardiac function is essential in elderly and comorbid patients undergoing Trz-based therapy for gastric cancer to mitigate the risk of cardiotoxicity.

**Conclusion:** Trz-based chemotherapy for HER2-positive gastric cancer, even without anthracyclines, may pose a risk of cardiotoxicity, particularly in elderly or comorbid patients. Further research is warranted to elucidate underlying mechanisms and optimize monitoring and prevention strategies in this population.

## 1. Introduction

Human epidermal growth factor receptor 2 (HER2) is a receptor tyrosine kinase encoded by the ErbB2 gene located on chromosome 17q21. It is part of the epidermal growth factor receptor (EGFR) family, which promotes cellular growth and proliferation [1, 2]. In cancer cells, HER2 gene overexpression or amplification is frequently observed and contributes to tumorigenesis. The prevalence of HER2 positivity (immunohistochemistry (IHC) 3+ or IHC 2+ with FISH positivity) in advanced or recurrent gastric cancer is reported to be approximately 15% [3].

Trastuzumab (Trz) is widely used as a first-line treatment for HER2-positive gastric cancer in combination with chemotherapy. The Trz for Gastric Cancer (ToGA) trial demonstrated the efficacy of this treatment [4]. Trz binds to the HER2 receptor, blocking downstream signaling pathways and inducing antibody-dependent cellular cytotoxicity (ADCC), thereby inhibiting cancer cell proliferation. This study showed that the combination of Trz with standard chemotherapy (cisplatin plus 5-fluorouracil or capecitabine) significantly improved overall survival (OS) compared with chemotherapy alone. Specifically, the median OS was 13.8 months in the Trz group compared with 11.1 months in the chemotherapy-only group.

Trz is associated with important adverse effects, including gastrointestinal symptoms, infusion reactions, and hematologic toxicity, in addition to cardiotoxicity [5]. The risk of cardiac dysfunction, particularly heart failure, increases when Trz is combined with anthracyclines for breast cancer treatment, necessitating regular cardiac monitoring during administration. However, reports of cardiovascular events occurring during chemotherapy for gastric cancer (without anthracycline use) are rare. Here, we report a case of HER2-positive gastric cancer in a postoperative patient who developed ventricular arrhythmia and heart failure during Trz-based chemotherapy for residual metastatic lymph nodes.

## 2. Case Presentation

An 80-year-old Japanese man with a 2-month history of abdominal pain and nausea was initially evaluated in the gastroenterology department. He had no history of dysphagia, hematemesis, or melena. His medical history included hypertension and lumbar spinal canal stenosis, with no known drug allergies. The patient had no history of alcohol consumption but had smoked 15 cigarettes per day for 20 years until he quit 20 years prior. His environmental and occupational history was unremarkable, and he had no family history of malignancies or cardiac disease.

### 2.1. Admission Findings

On admission, his height was 162.0 cm, weight was 65.0 kg, blood pressure was 122/64 mmHg, and pulse was 57 bpm. Physical examination revealed mild conjunctival pallor but no jaundice. Cardiac examination showed a regular heartbeat without murmurs, and pulmonary auscultation was clear. His abdomen was soft and nontender without distension, and no lower limb edema was observed.

### 2.2. Laboratory and Diagnostic Evaluations

Hematologic findings showed a white blood cell count of 8400 μL, hemoglobin of 7.8 g/dL, and platelet count of 405,000 μL. Blood biochemistry tests revealed a creatinine level of 0.67 mg/dL, blood urea nitrogen (BUN) of 11.0 mg/dL, total bilirubin of 0.8 mg/dL, aspartate aminotransferase (AST) of 18 IU/L, and alanine aminotransferase (ALT) of 9 IU/L. Tumor markers included carcinoembryonic antigen (CEA) at 66.0 ng/mL and carbohydrate antigen 19-9 (CA19-9) at < 2.0 U/mL. B-type natriuretic peptide (BNP) was 27.0 pg/mL.

**Figure 1:**
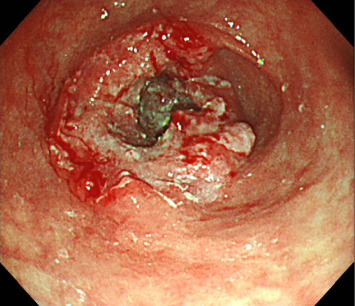
Esophagogastroduodenoscopy (EGD) revealed a circumferential type 2 tumor in the gastric antrum. A biopsy of the same site revealed a moderately differentiated adenocarcinoma.

**Figure 2:**
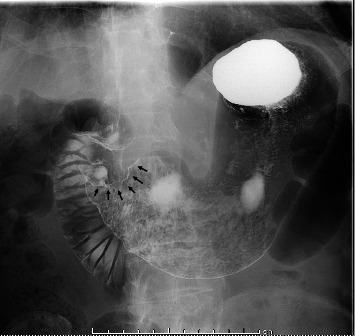
A gastric fluoroscopy revealed an irregular wall deformity in the antrum (arrow).

**Figure 3:**
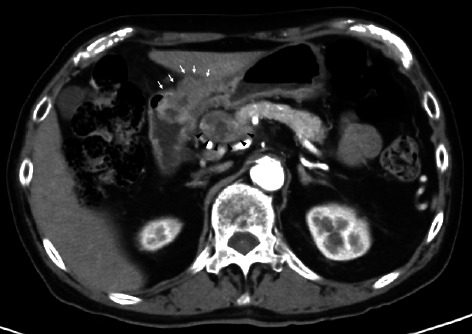
Contrast-enhanced imaging revealed circumferential wall thickening in the gastric antrum (arrow). Metastasis to the #8a lymph node and pancreatic invasion via the same lymph node were observed (arrowhead).

**Figure 4:**
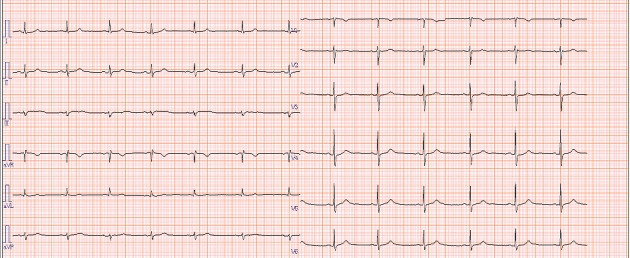
Preoperative electrocardiogram showing a heart rate of 55 bpm with sinus rhythm and no apparent waveform abnormalities.

Esophagogastroduodenoscopy (EGD) revealed a circumferential type 2 tumor in the gastric antrum (Figures 1, 2, and 3). Preoperative electrocardiography (ECG) showed no abnormalities (Figure 4), and echocardiography revealed a left ventricular ejection fraction (LVEF) of 60% (modified Simpson's method) without significant valvular disease, left ventricular hypertrophy, or wall motion abnormalities. The patient had an Eastern Cooperative Oncology Group (ECOG) performance status of 0. Due to gastric outlet obstruction and tumor bleeding-induced anemia, primary tumor resection and relief of stenosis via surgery were prioritized.

### 2.3. Surgical and Pathological Findings

**Figure 5:**
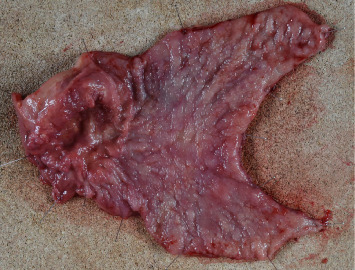
Pathological examination revealed a type 2 tumor measuring 65 × 60 mm with moderately differentiated tubular adenocarcinoma (tub2), no lymphatic or vascular invasion (ly0, v0), and tumor invasion into the subserosa (pT3). Six out of 49 dissected lymph nodes were positive for metastasis (pN2), and the final stage was pStage IIIA (the 15th edition of the Japanese gastric cancer treatment guidelines).

**Figure 6:**
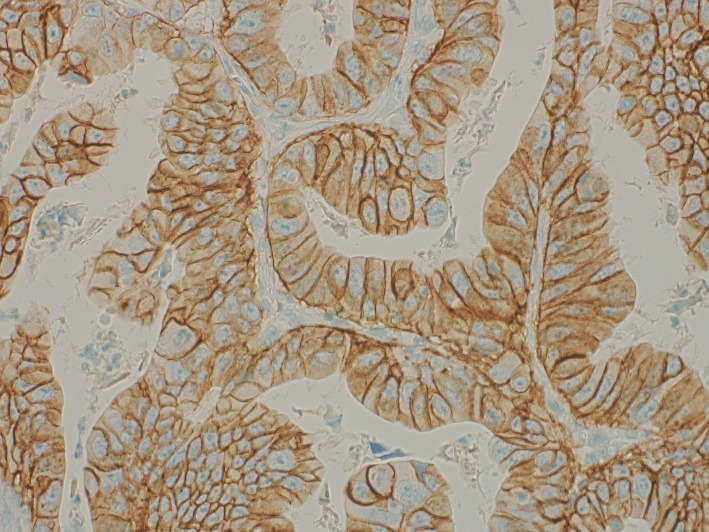
Immunohistochemical staining of the pathological specimen for HER2, showing 3+ positivity.

The patient was diagnosed with advanced gastric cancer (cT3N + M0, cStage III) and underwent open distal gastrectomy with D1+ lymph node dissection and Roux-en-Y reconstruction. Intraoperative peritoneal washing cytology was negative for tumor cells. However, intraoperative findings revealed lymph node metastasis in the anterior superior region of the common hepatic artery (#8a) and pancreatic invasion, prompting a strategy of adjuvant chemotherapy for residual disease. Pathological examination revealed a type 2 tumor measuring 65 × 60 mm with moderately differentiated tubular adenocarcinoma (tub2), no lymphatic or vascular invasion (ly0, v0), and tumor invasion into the subserosa (pT3). Six out of 49 dissected lymph nodes were positive for metastasis (pN2), and the final stage was pStage IIIA (the 15th edition of the Japanese Gastric Cancer Treatment Guidelines). Biomarker analysis of the resected specimen confirmed HER2 positivity (IHC 3+) and microsatellite stability (MSS) (Figures 5 and 6).

### 2.4. Chemotherapy and Clinical Course

Based on the ToGA trial [4], the patient was started on the Trz + SOX regimen. In the first cycle, Trz (8 mg/kg) was administered on Day 1, oxaliplatin (60 mg/m^2^) was given intravenously on Day 1, and TS-1 (100 mg/day) was administered orally from Days 1–14, followed by a 1 week recovery period. From the second cycle onwards, Trz (6 mg/kg) was given on Day 1, along with oxaliplatin (60 mg/m^2^) on Day 1 and TS-1 (100 mg/day) from Days 1–14, followed by a 1 week recovery period. Aprepitant (125 mg on Day 1 and 80 mg on Days 2 and 3) was given as premedication. After two cycles of Trz + SOX, computed tomography (CT) confirmed tumor shrinkage, and CEA levels declined to normal. Over 7 months of chemotherapy, a partial response (PR) was maintained per the Response Evaluation Criteria in Solid Tumors (RECIST). Serial echocardiograms performed every 3 months showed no significant cardiac dysfunction.

### 2.5. Adverse Cardiac Event

**Figure 7:**
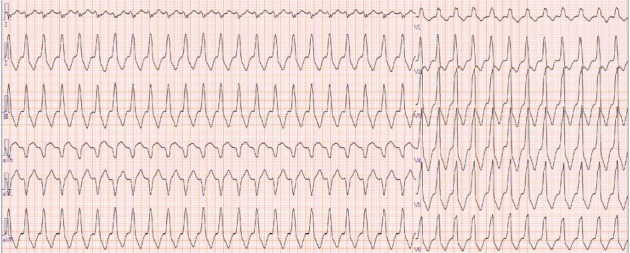
Electrocardiogram during an arrhythmic episode at the time of emergency admission, showing a heart rate of 138 bpm, ventricular tachycardia with wide QRS complexes, complete right bundle branch block, and right axis deviation.

**Figure 8:**
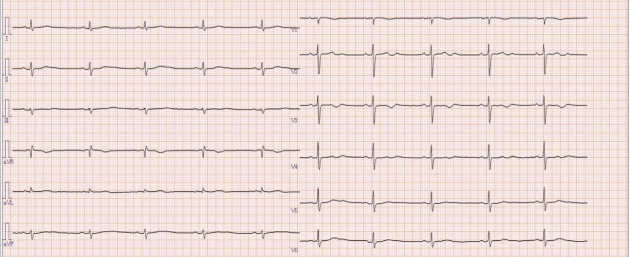
Electrocardiogram at discharge showing a heart rate of 43 bpm with sinus rhythm and no apparent waveform abnormalities.

Three days after the 11th cycle of Trz + SOX, the patient presented with chest pain, dyspnea, and syncope, prompting emergency admission. He was hypotensive (71/42 mmHg) with a bradycardia (42 bpm), no heart murmurs, and no lower limb edema. Laboratory tests showed mildly elevated high-sensitivity Troponin-I (38.7 pg/mL) and BNP (238.9 pg/mL), with normal thyroid function, normal C-reactive protein (CRP) level (0.14 mg/dL). The ECG during the chest pain attack revealed a heart rate of 138 bpm, ventricular tachycardia with a wide QRS, complete right bundle branch block, and right axis deviation (Figure 7). Chest radiography showed no cardiomegaly (cardiothoracic ratio 47%) or pulmonary congestion. Transthoracic echocardiography (TTE) demonstrated decreased cardiac contractility, an LVEF of 38%, and a reduced left ventricular global longitudinal strain (LV-GLS) of 17.7%. Coronary angiography excluded significant coronary artery disease, and cardiac MRI showed no myocardial thinning or enhancement. A decrease in LVEF and GLS led to a suspicion of Trz-induced cardiomyopathy (TIC). The patient was treated with amiodarone (initially 400 mg/day, then reduced to 150 mg/day due to QT prolongation), beta-blockers for frequent premature ventricular contractions (PVCs) and nonsustained VT (NSVT), and angiotensin receptor blockers (ARBs) for hypertension. His condition stabilized, and he was discharged on day 21. Serial 24-h electrocardiogram monitoring showed no runs of PVCs, and he remained asymptomatic (Figure 8).

### 2.6. Follow-Up and Current Status

Subsequently, the patient received TS-1 monotherapy (100 mg/day, Days 1–14, followed by a 1 week recovery period) for three months. Follow-up imaging confirmed the resolution of enlarged lymph nodes, indicating a complete response (CR). The patient continued TS-1 maintenance therapy without recurrence or arrhythmic events and has remained disease free for 2 years postoperatively.

## 3. Discussion

The ErbB family is one of the most extensively studied tyrosine kinase receptor families. In humans, four isoforms have been identified: ErbB1 (human epidermal growth factor receptor and HER1), ErbB2 (HER2), ErbB3 (HER3), and ErbB4 (HER4) [6, 7]. Under normal physiological conditions, these cell surface receptors mediate intercellular interactions, cell proliferation, and differentiation [1, 2]. Among the ErbB receptor family, HER2 is considered the most critical receptor as it plays an essential role in cell survival and is preferentially selected for dimerization with other receptor isoforms.

Trz is a recombinant humanized monoclonal antibody targeting the extracellular domain of the HER2 protein [8]. It is widely used as the first-line treatment for unresectable or advanced HER2-positive gastric cancer and as an adjuvant chemotherapeutic agent. Trz exerts its antitumor effect by binding to the HER2 receptor, inhibiting downstream signaling pathways, and inducing ADCC.

This case report describes a patient who developed a cardiac event during Trz-based chemotherapy for residual metastatic lymph nodes following surgery for HER2-positive gastric cancer. The patient exhibited significant tumor shrinkage with the Trz + SOX regimen but experienced a cardiac event characterized by ventricular tachycardia and heart failure symptoms during the 11th cycle of treatment. Despite guideline-recommended cardiac ultrasound evaluations every 3 months [9], no abnormalities such as left ventricular ejection fraction (LVEF) reduction were detected prior to the event. Cardiac imaging ruled out significant coronary artery disease and structural abnormalities, suggesting drug-induced myocardial injury. The patient was managed with antiarrhythmic therapy, and no recurrence of arrhythmia or symptoms was observed after discharge.

The underlying mechanism of TIC is thought to involve the inhibition of HER2 signaling in cardiomyocytes [10]. When HER2 signaling binds to neuregulin-1 (NRG-1), tyrosine kinase activation triggers the G-protein-coupled receptor signaling pathway. The G-protein alpha subunit activates the mitogen-activated protein kinase (MAPK) signaling pathway, leading to upregulation of extracellular signal regulated kinase (ERK) 1/2, a mediator that suppresses apoptosis (MAPK/ERK1/2-dependent pathway). In addition, NRG-1 signaling activates the phosphoinositide 3-kinase (PI3K)/serine/threonine kinase 1 (AKT) cascade, which alters mitochondrial respiration, reduces reactive oxygen species (ROS) production, and enhances cell survival (PI3K/AKT-dependent pathway). Furthermore, NRG-1 signaling activates focal adhesion kinase (FAK), an adapter protein of Src, which maintains sarcomere structure and function, promoting cardiomyocyte survival (FAK-dependent pathway). Collectively, these three signaling pathways help maintain stress responses, repair capacity, and prevent apoptosis under cardiac stress conditions [11, 12]. Trz binds to HER2 and inhibits HER2 signaling, suppressing these cardioprotective survival pathways. Since cardiomyocytes have high energy demands and require constant adenosine triphosphate (ATP) production, mitochondrial ATP generation is critical, making them prone to ROS accumulation. HER2 inhibition increases intracellular ROS levels, stimulating cardiomyocyte apoptosis and leading to heart failure [10–12]. Preclinical studies using HER2 knockout mouse models have demonstrated increased susceptibility to dilated cardiomyopathy and arrhythmias [13]. Studies using ErbB2 mutant mice have shown that the following cardiac toxicity phenotypes are observed: ① thinning of the ventricular wall, reduced LVEF, fibrosis, and increased myocardial cell apoptosis, leading to dilated cardiomyopathy (DCM) and death from heart failure within a few months [13] and ② progressive cardiac enlargement and reduced contractile function (LVEF < 40%), myocardial sarcomere structure breakdown, Z-line disorganization, increased cell death, and cardiac structural abnormalities due to fibroblast infiltration and interstitial fibrosis [14], among others. Similarly, studies using human cardiomyocytes have reported that inactivation of the erbB receptor causes changes in bcl-x splicing, promoting the formation of proapoptotic Bcl-xS, thereby contributing to increased apoptosis susceptibility in dysfunctional human cardiomyocytes [15]. The absence of coronary artery disease, combined with cardiac imaging findings suggesting myocardial fibrosis in this case, aligns with previous histopathological reports of TIC showing myocardial injury without inflammatory infiltration [16]. In addition, risk factors such as advanced age, hypertension, and anemia may have contributed to the patient's vulnerability. Literature suggests that elderly patients are at increased risk of cardiotoxicity due to age-related declines in cardiac reserve [17]. In addition, oxaliplatin, though less commonly implicated in cardiac toxicity, has been associated with transient arrhythmias, oxidative stress, and mitochondrial dysfunction in cardiomyocytes [18, 19]. The concomitant administration of Trz and oxaliplatin may, therefore, have a synergistic effect on cardiac toxicity via shared mechanisms involving ROS accumulation and calcium dysregulation [20]. Further investigation is needed to clarify this potential interaction.

Recent guidelines by the European Society of Cardiology and the International Cardio-Oncology Society emphasize the need for baseline and longitudinal cardiac assessment in patients undergoing HER2-targeted therapy [9]. In cardiac oncology, TTE is the first choice for cardiac surveillance in tumor patients because it does not involve radiation exposure and is highly cost-effective, making it ideal for continuous surveillance. Compared with continuous LVEF monitoring, frequent evaluation of LV-GLS is recommended to detect cancer therapy-related cardiac dysfunction (CTRCD) early during Trz chemotherapy and to promptly initiate medical treatment for heart failure [21]. However, the evaluation of LVEF using 2D TTE has been reported to have interobserver and intraobserver variability of 10% over time, and cardiac magnetic resonance imaging (CMR) is increasingly playing a crucial role in cardiac oncology [22]. Single-photon emission computed tomography/computed tomography (SPECT/CT) scans using 99mTc have also been reported to be useful for early LVEF evaluation [23]. Circulating biomarkers of myocardial injury may offer even earlier and more sensitive detection of subclinical cardiac dysfunction than imaging modalities. Troponin-I and Brain Natriuretic Peptide (BNP), the marker of myocardial cell injury, tends to be elevated in patients receiving anti-HER2 therapy following prior chemotherapy for breast cancer, indicating a higher risk of cardiac dysfunction [24].

**Figure 9:**
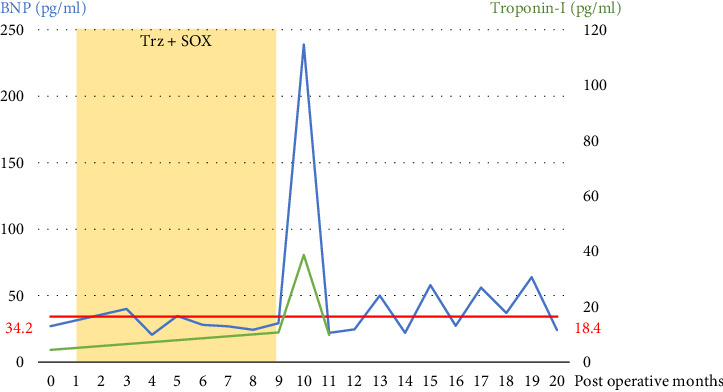
Serial changes in BNP and troponin-I levels measured during trastuzumab-based chemotherapy (Trz + SOX). A transient elevation was observed at the time of the BNP peak: 238.9 pg/mL, troponin-I peak: 38.7 pg/mL. Noted event: trastuzumab-induced cardiotoxicity (TIC) onset. Episode of heart failure (postoperative month 9).

In this case, BNP, a biomarker of cardiac toxicity, was measured at each cycle of chemotherapy. BNP increased from below the reference range to 238.9 pg/mL during a -I was measured before surgery, before heart failure episode, during episode, and 1 month after episode, but only showed a mild increase during the episode (Figure 9). CRP, an inflammatory marker associated with myocarditis, did not exceed 0.50 mg/dL. Elevated BNP and Troponin-I levels were observed only during the acute phase of heart failure. Neither of the blood test results provided any indication that heart failure would occur. Serial cardiac monitoring was conducted throughout the treatment period. Findings from 24-h electrocardiogram monitoring are described (Table 1). At onset, frequent PVCs, accounting for approximately 9.5% of total beats, including bigeminy and trigeminy, were also frequent and persisted for a prolonged period. With the use of antiarrhythmic drugs, the number of PVCs and ventricular arrhythmia events were controlled. No significant changes were observed in atrial arrhythmias. Evaluate the findings of the cardiac echocardiogram (Table 2). TTE demonstrated a significant decline in left ventricular systolic function following the heart failure episode. The LVEF decreased from 69% to 38%, and fractional shortening (FS) dropped from 42% to 17%. There was a notable increase in both LV end-diastolic (94 mL–126 mL) and end-systolic volumes (29 mL–42 mL), indicating progressive ventricular dilation. In addition, LV-GLS measured post-episode was reduced to −17.7%, supporting a decline in myocardial deformation capacity. Right ventricular function also declined markedly, with tricuspid annular plane systolic excursion (TAPSE) falling from 25.1 mm to 14.4 mm, and S′ velocity decreasing from 12 cm/s to 5.9 cm/s. Diastolic filling parameters revealed a shift toward a restrictive pattern, with the *E*/*A* ratio rising from 1.3 to 3.4 and persistently elevated *E*/*e*' ratios (16-17), suggesting increased left atrial pressure. No significant valvular abnormalities or progression were observed. These findings collectively indicate the development of biventricular dysfunction caused by TIC.

In this case, after a cardiovascular event, Trz was discontinued following current clinical guidelines for patients with LVEF reduction [9]. The SAFE-HEaRt trial reported the cardiac safety of initiating anti-HER2 therapy in asymptomatic patients with mildly reduced LVEF (40%–49%) [25]. However, no study has evaluated the resumption or continuation of anti-HER2 therapy in patients experiencing cardiovascular events. Hussain reported that among patients with LVEF < 50% who continued anti-HER2 therapy, 21 cases (91%) received new or intensified cardiovascular therapy (beta-blockers, angiotensin-converting enzyme inhibitors, and/or ARB) under cardiologist supervision, with 14 cases (61%) tolerating Trz without cardiovascular events, 6 cases (26%) experiencing asymptomatic LVEF decline leading to permanent discontinuation, and 3 cases (13%) developing cardiovascular events [26]. For the treatment of ventricular arrhythmias in patients with heart failure and reduced left ventricular ejection fraction, amiodarone is selected due to its efficacy and low proarrhythmic effect [27]. In addition, in rats with dilated cardiomyopathy, long-term administration of amiodarone has been reported to suppress left ventricular remodeling and improve cardiac function [28]. Given the significant survival benefits of Trz-containing regimens [5], continuation of Trz might be considered even after asymptomatic LVEF decline, particularly in high-risk cancer patients. However, the decision to continue Trz must be made after a careful assessment of both cardiovascular and oncological risks and benefits. Collaboration between cardiologists and oncologists is essential for optimizing cardiac management and monitoring. Further studies are needed to identify predictors of cardiovascular outcomes in patients who develop cardiovascular events during Trz-based therapy.

This report has inherent limitations as it describes a single case, and incidental cardiotoxicity or electrocardiographic abnormalities cannot be entirely ruled out. Moreover, the potential influence of the patient's medical history, underlying conditions, and concomitant medications remains a confounding factor. Consequently, establishing a definitive causal relationship between the observed cardiotoxicity and Trz monotherapy—rather than a synergistic effect from combination chemotherapy—remains challenging. Nonetheless, this case underscores the importance of close cardiac monitoring even in patients without prior cardiac history.

## 4. Conclusion

Currently, no standardized guidelines exist for the resumption of Trz after a cardiovascular event. While recent studies suggest that some patients with mildly reduced LVEF can tolerate continued HER2-targeted therapy with appropriate cardioprotective measures, further research is needed to establish predictors of TIC and determine individualized management strategies. Close collaboration between oncologists and cardiologists is essential to balance oncological efficacy with cardiovascular safety in patients undergoing HER2-targeted therapy for gastric cancer.

## Figures and Tables

**Table 1:** Changes in 24-h electrocardiogram (ECG) monitoring findings after TIC.

Parameter	At onset of TIC	After treatment	Interpretation
PVC burden (% of total beats)	9.5%	< 1%	Controlled with amiodarone
Pattern	Bigeminy, trigeminy	None observed	Ventricular ectopy resolved
NSVT episodes	Frequent	None	Suppressed
QTc interval (ms)	475	440	Mild prolongation, improved
Supraventricular arrhythmias	No change	No change	Stable
Tachycardia (> 140 bpm)	Present	Absent	Resolved
Bradycardia (< 40 bpm)	Present (discharge)	Absent	Resolved

*Note:* At the onset of TIC, the patient exhibited a high PVC burden with complex patterns including bigeminy and trigeminy. Antiarrhythmic therapy led to marked improvement in all ventricular arrhythmias, while supraventricular arrhythmias remained unchanged.

Abbreviations: AF = atrial fibrillation, NSVT = nonsustained ventricular tachycardia, PVC = premature ventricular contraction, SVC = supraventricular contraction.

**Table 2:** Changes in transthoracic echocardiography (TTE) findings before and after TIC.

Parameter	Pre-TIC	Post-TIC	Interpretation
LVEF (%)	69	38	Marked reduction in systolic function
FS (%)	42	17	Progressive contractile dysfunction
LVEDV (mL)	94	126	Increase in end-diastolic volume
LVESV (mL)	29	42	Increased end-systolic volume
LV-GLS (%)	—	−17.7	Decreased myocardial deformation
TAPSE (mm)	25.1	14.4	Right ventricular dysfunction
*S*' velocity (cm/s)	12	5.9	Impaired RV systolic motion
*E*/*A* ratio	1.3	3.4	Shift to restrictive LV filling pattern
*E*/*e*' ratio	16	17	Elevated LV filling pressure

*Note:* Echocardiographic parameters before and after the onset of TIC demonstrated significant deterioration in both left and right ventricular function. Left ventricular ejection fraction (LVEF) and fractional shortening (FS) markedly declined, accompanied by increased end-diastolic and end-systolic volumes. Global longitudinal strain (GLS) and tricuspid annular plane systolic excursion (TAPSE) also worsened. Diastolic parameters shifted toward a restrictive filling pattern, suggesting elevated left atrial pressure and biventricular dysfunction.

Abbreviations: FS = fractional shortening, LVEDV = LV end-diastolic volume, LVEF = left ventricular ejection fraction, LVESV = LV end-systolic volume, LV-GLS = LV global longitudinal strain, TAPSE = tricuspid annular plane systolic excursion, TIC = trastuzumab-induced cardiotoxicity.

## Data Availability

The data that support the findings of this study are available from the corresponding author upon reasonable request.

## References

[1] Ménard S., Pupa S. M., Campiglio M., Tagliabue E. (2003). Biologic and Therapeutic Role of HER2 in Cancer. *Oncogene*.

[2] Rubin I., Yarden Y. (2001). The Basic Biology of HER2. *Annals of Oncology*.

[3] Japanese Gastric Cancer Association (2025). Japanese Gastric Cancer Treatment Guidelines 2025. *Gastric Cancer*.

[4] Bang Y., Van C. E., Feyereislova A. (2010). Trastuzumab in Combination With Chemotherapy Versus Chemotherapy Alone for Treatment of HER2-Positive Advanced Gastric or Gastroesophageal Junction Cancer (Toga): A Phase 3, Open-Label, Randomised Controlled Trial. *Lancet*.

[5] Leocachin-Parra D. L., Bocardo-Galvan J. B., Grajales R., Carrillo-Estrada M. (2024). Trastuzumab-Induced Cardiotoxicity in Patients With Metastatic HER2-positive Breast Cancer Treated at a Reference Hospital in Mexico. *Journal of Clinical Orthodontics*.

[6] Feyen E., Ricke-Hoch M., Van Fraeyenhove J. (2021). ERBB4 and Multiple Micrornas That Target ERBB4 Participate in Pregnancy-Related Cardiomyopathy. *Circulation: Heart Failure*.

[7] Qiang Z., Wan J., Chen X., Wang H. (2024). Mechanisms and Therapeutic Targets of Erbb Family Receptors in Hepatocellular Carcinoma: A Narrative Review. *Translational Cancer Research*.

[8] Zhang X., Yin Y., Yu Q., Chen X., Cheng Y. (2024). Review of the Clinical Status of Cardiotoxicity of HER-2 Positive Breast Cancer Targeted Therapeutic Drugs. *Frontiers in Oncology*.

[9] Lyon A. R., Dent S., Stanway S. (2020). Baseline Cardiovascular Risk Assessment in Cancer Patients Scheduled to Receive Cardiotoxic Cancer Therapies: A Position Statement and New Risk Assessment Tools From the Cardio-Oncology Study Group of the Heart Failure Association of the European Society of Cardiology in Collaboration With the International Cardio-Oncology Society. *European Journal of Heart Failure*.

[10] Wu Q., Bai B., Tian C. (2022). The Molecular Mechanisms of Cardiotoxicity Induced by HER2, VEGF, and Tyrosine Kinase Inhibitors: An Updated Review. *Cardiovascular Drugs and Therapy*.

[11] Eaton H., Timm K. N. (2023). Mechanisms of Trastuzumab Induced Cardiotoxicity-Is Exercise a Potential Treatment?. *Cardio-Oncology*.

[12] Sabbatino F., Conti V., Liguori L. (2021). Molecules and Mechanisms to Overcome Oxidative Stress Inducing Cardiovascular Disease in Cancer Patients. *The Life*.

[13] Crone S. A., Zhao Y. Y., Fan L. (2002). ErbB2 is Essential in the Prevention of Dilated Cardiomyopathy. *Nature Medicine*.

[14] Ozcelik C., Erdmann B., Pilz B. (2002). Conditional Mutation of the ErbB2 (HER2) Receptor in Cardiomyocytes Leads to Dilated Cardiomyopathy. *Proceedings of the National Academy of Sciences of the United States of America*.

[15] Rohrbach S., Niemann B., Silber R. E., Holtz J. (2005). Neuregulin Receptors erbB2 and erbB4 in Failing Human Myocardium-Depressed Expression and Attenuated Activation. *Basic Research in Cardiology*.

[16] Russell S. D., Blackwell K. L., Lawrence J. (2010). Independent Adjudication of Symptomatic Heart Failure With the Use of Doxorubicin and Cyclophosphamide Followed by Trastuzumab Adjuvant Therapy: A Combined Review of Cardiac Data From the National Surgical Adjuvant Breast and Bowel Project B-31 and the North Central Cancer Treatment Group N9831 Clinical Trials. *Journal of Clinical Oncology*.

[17] Albini A., Pennesi G., Donatelli F., Cammarota R., De Flora S., Noonan D. M. (2010). Cardiotoxicity of Anticancer Drugs: The Need for Cardio-Oncology and Cardio-Oncological Prevention. *JNCI Journal of the National Cancer Institute*.

[18] Adhikari A., Asdaq S. M. B., Al Hawaj M. A. (2021). Anticancer Drug-Induced Cardiotoxicity: Insights and Pharmacogenetics. *Pharmaceuticals*.

[19] Tocchetti C. G., Cadeddu C., Di Lisi D. (2019). From Molecular Mechanisms to Clinical Management of Antineoplastic Drug-Induced Cardiovascular Toxicity: A Translational Overview. *Antioxidants and Redox Signaling*.

[20] Gondal M. U. R., Lemoine J., Segal J. (2024). Cardiotoxicity Induced by Capecitabine and Oxaliplatin in Gastric Cancer Treatment: A Rare Case of Cardiac Arrest and Cardiogenic Shock. *European journal of case reports in internal medicine*.

[21] Nhat G. M., Hai N. H., Duc V. T., Tri H. H. Q., Hoa C. N. (2024). Features of Trastuzumab-Related Cardiac Dysfunction: Deformation Analysis Outside Left Ventricular Global Longitudinal Strain. *Frontiers in Cardiovascular Medicine*.

[22] Jiang J., Liu B., Hothi S. S. (2022). Herceptin-Mediated Cardiotoxicity: Assessment by Cardiovascular Magnetic Resonance. *Cardiology Research and Practice*.

[23] Gherghe M., Lazar A. M., Mutuleanu M. (2022). Evaluating Cardiotoxicity in Breast Cancer Patients Treated With HER2 Inhibitors: Could a Combination of Radionuclide Ventriculography and Cardiac Biomarkers Predict the Cardiac Impact?. *Cancers*.

[24] Cardinale D., Iacopo F., Cipolla C. M. (2020). Cardiotoxicity of Anthracyclines. *Frontiers in Cardiovascular Medicine*.

[25] Lynce F., Barac A., Geng X. (2019). Prospective Evaluation of the Cardiac Safety of HER2-targeted Therapies in Patients With HER2-Positive Breast Cancer and Compromised Heart Function: The SAFE-HEaRt Study. *Breast Cancer Research and Treatment*.

[26] Hussain Y., Drill E., Dang C. T., Liu J. E., Steingart R. M., Yu A. F. (2019). Cardiac Outcomes of Trastuzumab Therapy in Patients With HER2-Positive Breast Cancer and Reduced Left Ventricular Ejection Fraction. *Breast Cancer Research and Treatment*.

[27] Pannone L., D’Angelo G., Gulletta S. (2021). Amiodarone in Ventricular Arrhythmias: Still a Valuable Resource?. *Reviews in Cardiovascular Medicine*.

[28] Tachikawa H., Kodama M., Watanabe K. (2005). Amiodarone Improves Cardiac Sympathetic Nerve Function to Hold Norepinephrine in the Heart, Prevents Left Ventricular Remodeling, and Improves Cardiac Function in Rat Dilated Cardiomyopathy. *Circulation*.

